# Buyang Huanwu Decoction Modulates the Gut Microbiota–C/EBPβ/AEP Axis to Ameliorate Cognitive Impairment in Alzheimer's Disease Mice

**DOI:** 10.1111/cns.70480

**Published:** 2025-06-23

**Authors:** Junyi Liang, Xiaohong Dong, Jianshe Yang, Niyuan Hu, Xiaoting Luo, Shuyuan Cong, Jing Chen, Weiming Zhao, Bin Liu

**Affiliations:** ^1^ Heilongjiang University of Traditional Chinese Medicine Harbin Heilongjiang Province China; ^2^ Heilongjiang Jiamusi Central Hospital Jiamusi Heilongjiang Province China; ^3^ Harbin Sport University Harbin Heilongjiang Province China; ^4^ Yunnan University of Chinese Medicine Kunming Yunnan Province China

**Keywords:** AEP, Alzheimers disease, amyloid‐β, Buyang Huanwu decoction, C/EBPβ, gut microbiota

## Abstract

**Background:**

Alzheimer's disease (AD) is a progressive neurodegenerative disorder characterized by cognitive decline and behavioral disturbances. Buyang Huanwu Decoction (BYHWD), a traditional Chinese herbal formulation, has demonstrated potential neuroprotective effects. This study aims to evaluate the therapeutic impact of BYHWD on cognitive impairments in 3×Tg mice and to investigate its underlying mechanism through modulation of the gut microbiota–C/EBPβ/AEP signaling pathway.

**Methods:**

In two independent experiments, we assessed the effects of BYHWD and its derived fecal microbiota transplantation (FMT‐BYHWD) on behavioral performance, neuropathological alterations, and signaling pathways in 3×Tg mice.

**Results:**

Treatment with BYHWD significantly improved cognitive function in 3×Tg mice and mitigated AD‐like pathological changes. By suppressing the C/EBPβ/AEP signaling pathway, BYHWD reduced pathological Aβ plaque deposition, diminished tau hyperphosphorylation, and inhibited the release of pro‐inflammatory cytokines. Further analysis revealed that BYHWD restored gut microbiota balance and suppressed the activation of the C/EBPβ/AEP pathway in the hippocampus. Moreover, transplanting FMT‐BYHWD from BYHWD‐treated mice to germ‐free 3×Tg mice also ameliorated their cognitive deficits and AD‐like pathology, suggesting that the anti‐AD effects of BYHWD are mediated through the gut–brain axis by regulating the interplay between gut microbiota and the C/EBPβ/AEP signaling pathway.

**Conclusion:**

This study uncovers the mechanism by which BYHWD improves cognitive deficits and neuropathological changes in 3×Tg mice via the gut–brain axis, mediated by the modulation of the gut microbiota‐C/EBPβ/AEP signaling pathway, providing a novel therapeutic strategy for AD.

## Graphical abstract

Our study demonstrates that BYHWD ameliorates Alzheimer's disease pathology by modulating the gut microbiota and suppressing the C/EBPβ–AEP signaling pathway. BYHWD reverses gut dysbiosis, leading to downregulation of C/EBPβ and reduced AEP‐mediated cleavage of APP and Tau into APP N585 and Tau N368, respectively. This cascade attenuates Aβ accumulation, Tau hyperphosphorylation, and neuroinflammation, thereby improving cognitive function in 3×Tg mice.

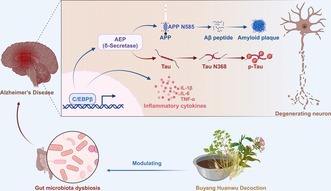

## 1 Introduction

Alzheimer's disease (AD) is a progressive neurodegenerative disorder characterized by cognitive decline, synaptic dysfunction, and pathological accumulation of amyloid‐β (Aβ) and hyperphosphorylated tau [1, 2]. While Aβ accumulation and tau hyperphosphorylation are hallmarks of AD, emerging evidence suggests that gut microbiota dysbiosis may play a fundamental role in disease progression via the microbiota–gut–brain axis [3, 4, 5]. Changes in gut microbial composition have been linked to neuroinflammation, altered immune responses, and blood–brain barrier integrity, all of which are implicated in AD pathology [5, 6, 7]. However, the mechanistic relationship between gut microbiota and AD pathogenesis remains to be fully elucidated.

Aspartyl endopeptidase (AEP, gene name *LGMN*), also known as δ‐secretase, plays a pivotal role in AD pathogenesis by cleaving amyloid precursor protein (APP) and tau, thereby promoting Aβ generation and tau hyperphosphorylation [8]. Inhibition of AEP in transgenic AD mouse models alleviates Aβ and tau pathology, restoring synaptic function and cognitive performance [8, 9, 10]. Upstream, CCAAT/enhancer‐binding protein β (C/EBPβ) transcriptionally regulates AEP in an age‐dependent manner, amplifying neuroinflammatory responses and accelerating AD progression [11]. Overexpression of C/EBPβ enhances AEP expression, accelerating AD pathology and exacerbating cognitive deficits in young 3×Tg mice. Activation of C/EBPβ triggers AEP‐mediated cleavage of APP N585 and tau N368, promoting Aβ production and tau hyperphosphorylation, thereby inducing neuroinflammation and neurotoxicity, which drive AD pathogenesis [11, 12, 13]. Thus, the C/EBPβ/AEP signaling axis thus represents a key pathological pathway in AD, yet its regulation via extrinsic factors, such as gut microbiota, remains largely unexplored.

Traditional Chinese medicine (TCM) has attracted growing interest as a potential therapeutic strategy for AD, with Buyang Huanwu Decoction (BYHWD) being particularly noted for its reported neuroprotective effects. BYHWD, a classical TCM formula, originates from *Yi Lin Gai Cuo*, an ancient medical compendium by Wang Qingren during the Qing Dynasty [14]. This formulation comprises seven herbal ingredients: *Astragalus mongholicus Bunge* (Huang Qi), *Angelica sinensis* (Oliv.) Diels (Dang Gui Wei), *Paeonia veitchii Lynch* (Chi Shao), *Ligusticum chuanxiong* (Chuan Xiong), *Prunus persica* (L.) Batsch (Tao Ren), *Carthamus tinctorius* L. (Hong Hua), and *Pheretima aspergillum* (E. Perrier) (Di Long), and has been reported to improve neurological function by promoting blood circulation and neuroprotection [14]. Our previous studies demonstrated that BYHWD alleviates cognitive impairment in APP/PS1 mice [15]. Additionally, BYHWD has been shown to modulate gut microbiota, conferring protective effects against gastrointestinal disorders [16, 17]. However, whether BYHWD exerts its neuroprotective effects in AD via the microbiota–gut–brain axis and the C/EBPβ/AEP pathway remains unknown.

Here, we investigate the therapeutic potential of BYHWD in the 3×Tg mouse model, which recapitulates key features of Aβ accumulation and tau hyperphosphorylation. In addition to these pathological hallmarks, 3×Tg mice exhibit neuronal loss, synaptic dysfunction, and gut microbiota dysbiosis, alongside activation of the C/EBPβ/AEP signaling pathway [11, 18]. We examine whether BYHWD mitigates cognitive impairment by reshaping gut microbiota and modulating the C/EBPβ/AEP axis, thereby offering mechanistic insights into microbiota‐targeted strategies for AD intervention.

## 2 Materials and Methods

### 2.1 Animals and Reagents

A total of 64 SPF‐grade male 3×Tg and 16 C57BL/6 mice (2–3 months old) were used and maintained under standard conditions. All animal procedures were approved by the Animal Ethics Committee of Heilongjiang University of Chinese Medicine. BYHWD and donepezil were administered according to previous reports. The dosing regimen for BYHWD (9.26 and 37.05 g/kg/day) and donepezil (5 mg/kg/day) was determined based on our preliminary experimental findings and corroborated by published literature (see Section 2.3). Antibodies, antibiotics, ELISA kits, staining reagents, and PCR primers were commercially obtained. Detailed information on animal sourcing, herbal preparation, and reagent specifications is provided in Methods S1.

### 2.2 Preparation of BYHWD

BYHWD was prepared from seven herbs according to traditional ratios, decocted, concentrated, and freeze‐dried into powder. The final extract was stored at 4°C and reconstituted in water prior to administration. Detailed preparation procedures and yield information are available in Methods S1.

### 2.3 Animal Grouping and Administration

**FIGURE 1:**
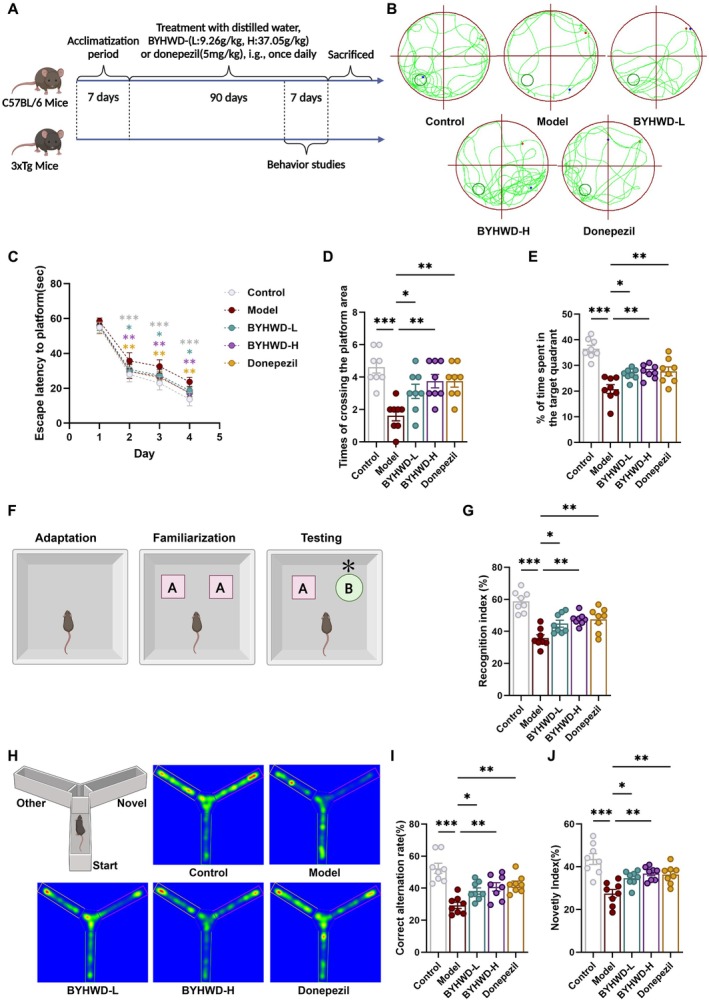
BYHWD ameliorates behavioral deficits and cognitive impairments in 3×Tg mice. (A) Experimental timeline and schematic illustrating the first phase of the study. (B) Representative swimming trajectories from the Morris water maze task, used to assess spatial learning and memory in 3×Tg mice. (C) Escape latency across the four training days (Days 1–4) in the Morris water maze test, reflecting spatial memory acquisition. Data are shown as mean ± SD (*n* = 8). (D) Number of platform crossings during the probe trial of the Morris water maze, indicating memory retention and the ability to recall the learned platform location. (E) Time spent in the target quadrant during the probe trial, reflecting spatial memory performance, and retention. (F) Schematic of the novel object recognition test, assessing non‐spatial memory in 3×Tg mice. (G) Recognition index calculated from the time spent exploring the novel object versus the familiar object during the novel object recognition test. (H) Representative heatmaps showing exploration behavior in the Y‐maze, reflecting preference for the novel arm as an indicator of working memory and cognitive flexibility. (I) Percentage of correct alternations in the Y‐maze, measuring working memory and cognitive flexibility. (J) Novelty index in the Y‐maze, quantifying preference for the novel arm as an indicator of exploratory behavior and memory. Data are presented as mean ± SEM (*n* = 8) unless otherwise specified. Normality was assessed using the Shapiro–Wilk test, and variance homogeneity was tested by Bartlett's test. Statistical significance was assessed using one‐way ANOVA with Dunnett's post hoc test (D, E, G, I, J) and two‐way ANOVA with Bonferroni's post hoc test (C). Statistical significance was determined as *p < 0.05; **p < 0.01; ***p < 0.001.

Experiment 1: 3×Tg mice were randomly assigned to four groups (*n* = 8/group): Model group (3×Tg mice receiving distilled water only); BYHWD‐L group (3×Tg mice receiving low‐dose BYHWD at 9.26 g/kg/day); BYHWD‐H group (3×Tg mice receiving high‐dose BYHWD at 37.05 g/kg/day); Donepezil group (3×Tg mice treated with Donepezil at 5 mg/kg/day). Age‐matched wild‐type C57BL/6 mice served as the Control group (receiving distilled water only). All treatments were administered by daily gavage for 90 days. The dosing regimen for BYHWD was determined based on our preliminary studies and corroborated by published literature (see Methods S1). After 90 days of treatment and behavioral evaluation, mice were euthanized for tissue collection (Figure 1A).

Experiment 2: To evaluate the role of gut microbiota in the therapeutic effects of BYHWD, fecal microbiota transplantation (FMT) was performed. 3×Tg mice were divided into four groups (*n* = 8/group): M + Vehicle group (3×Tg mice receiving vehicle only); M + FMT‐C group (3×Tg mice receiving FMT from Control mice); M + FMT‐M group (3×Tg mice receiving FMT from Model mice); and M + FMT‐BYHWD group (3×Tg mice receiving FMT from BYHWD‐H treated mice). All mice were pretreated with a cocktail of antibiotics (neomycin, ampicillin, metronidazole) for 7 days to induce a pseudo‐germ‐free state. FMT was performed using fecal suspensions (50 mg/mL, 0.6 mL/day) collected from Control, Model, and BYHWD‐H mice from Experiment 1. After 90 days of treatment and behavioral evaluation, mice were euthanized for tissue collection (Figure 5A).

### 2.4 Behavioral Testing

Cognitive performance was assessed using the Morris water maze, novel object recognition, and Y‐maze tests. Detailed protocols are available in Methods S1.

### 2.5 Sample Collection

Mice were anesthetized and perfused with PBS. Brains were either fixed in 4% paraformaldehyde or snap‐frozen. Hippocampal tissues and fecal samples were stored at −80°C for subsequent analysis. Full procedures are detailed in Methods S1.

### 2.6 Pathological Staining

Brain tissues were extracted, fixed in 4% paraformaldehyde, and processed for histological analysis. Sections were stained with hematoxylin and eosin (HE) and Nissl methods, followed by examination and imaging under an optical microscope.

### 2.7 Enzyme‐Linked Immunosorbent Assay

Levels of Aβ40, Aβ42, IL‐1β, IL‐6, and TNF‐α in hippocampal homogenates were measured using commercial ELISA kits according to the manufacturer's instructions.

### 2.8 Western Blot Analysis

Western blotting was performed to assess the expression of C/EBPβ, AEP, APP NT, APP N585, Tau5, Tau N368, p‐Tau (T205, S396), and β‐actin in hippocampal tissue. Detailed procedures are provided in Methods S1.

### 2.9 Immunohistochemistry

Immunohistochemical analysis was conducted to evaluate the expression of phosphorylated Tau protein in brain sections. Detailed staining procedures, including antigen retrieval, antibody incubation, and signal development, are provided in Methods S1.

### 2.10 Immunofluorescence Staining

Immunofluorescence staining was performed on brain sections to detect Aβ and C/EBPβ expression. The complete protocol, including section preparation, antibody information, and image analysis, is available in Methods S1.

### 2.11 qPCR Analysis

Quantitative PCR was conducted to evaluate the mRNA expression levels of C/EBPβ (*Cebpb*) and AEP (*Lgmn*) in hippocampal tissue. Details of RNA extraction, cDNA synthesis, primer sequences, and amplification conditions are provided in Methods S1.

### 2.12 16S rRNA Gene Sequencing

Fecal microbiota composition was analyzed through 16S rRNA gene sequencing targeting the V3–V4 hypervariable regions. Sequencing was conducted on the Illumina NovaSeq platform, and data were analyzed using QIIME2 (v2022.11) for taxonomic classification, α‐diversity, and PCoA. Detailed protocols are provided in Methods S1.

### 2.13 Statistical Analysis

All statistical analyses were performed using GraphPad Prism 10. Data are presented as mean ± standard error of the mean (SEM). The normality of data distribution was assessed using the Shapiro–Wilk test. For normally distributed data, comparisons among multiple groups were made using one‐way analysis of variance (ANOVA) followed by Dunnett's post hoc test. For non‐normally distributed data, nonparametric analyses (Kruskal–Wallis test) were applied. Morris water maze training data (escape latency across days) were analyzed using two‐way ANOVA with Bonferroni's post hoc test. A *p*‐value of < 0.05 was considered statistically significant.

## 3 Results

### 3.1 BYHWD Modulates the Gut Microbiota‐C/EBPβ/AEP Axis to Ameliorate the Pathological State of 3×Tg Mice

#### 3.1.1 BYHWD Ameliorates Learning and Memory Deficits in 3×Tg Mice

The 3×Tg mice manifest dementia‐like symptoms, including cognitive and memory dysfunctions [19, 20]. In the current set of experiments, we utilized the Morris water maze to evaluate the spatial learning and memory abilities of the mice. During the training phase, the model group displayed prolonged escape latency compared to the control group, indicating impaired spatial learning. In contrast, the donepezil, BYHWD‐L, and BYHWD‐H groups exhibited shorter escape latencies, suggesting enhanced learning efficiency (Figure 1C). In the probe trial, the model group showed fewer platform crossings and reduced dwell time in the target quadrant, reflecting memory retention deficits. Notably, these impairments were significantly alleviated in the donepezil and BYHWD‐H groups, while the BYHWD‐L group showed moderate improvement (Figure 1B–E).

To assess recognition memory, we conducted the Novel Object Recognition Test (Figure 1F), which leverages the natural tendency of mice to explore novel objects over familiar ones. The model group exhibited a markedly lower recognition index than the control group, indicating impaired object discrimination. Notably, the donepezil and BYHWD‐H groups showed significant improvements in recognition memory, while the BYHWD‐L group exhibited a moderate enhancement (Figure 1G).

Spatial learning and working memory were further evaluated using the Y‐maze test. The model group displayed reduced spontaneous alternation and novelty preference indices, reflecting deficits in cognitive flexibility and spatial memory. In contrast, the donepezil and BYHWD‐H groups showed substantial improvements, whereas the BYHWD‐L group demonstrated moderate enhancement (Figure 1H–J). Collectively, these findings indicate that BYHWD mitigates learning and memory deficits in 3×Tg mice.

#### 3.1.2 BYHWD Suppresses the Overactivated C/EBPβ/AEP Signaling Pathway in 3×Tg Mice

**FIGURE 2:**
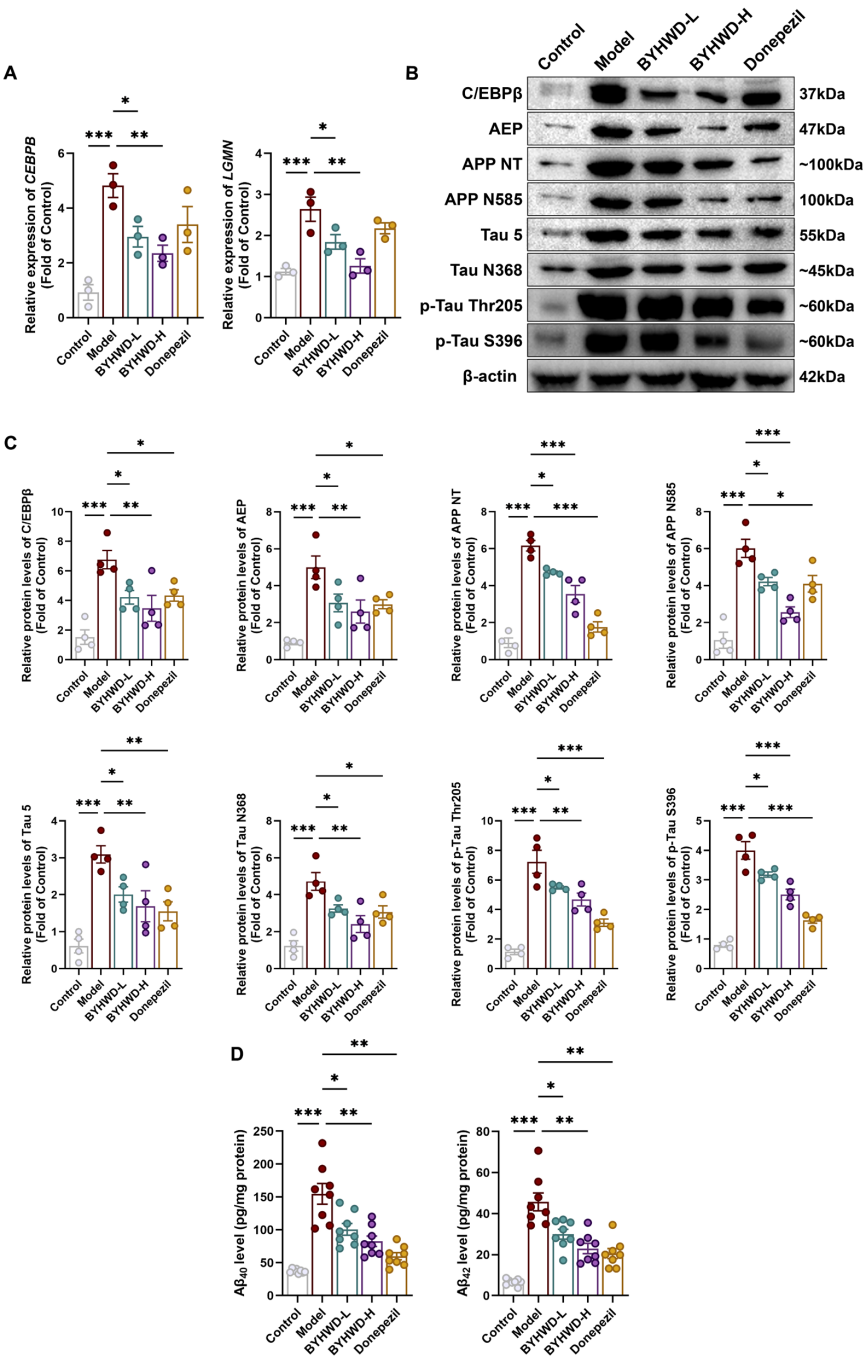
BYHWD suppresses the C/EBPβ‐AEP pathway and reduces Aβ burden in 3×Tg mice. (A) qPCR analysis of hippocampal *Cebpb* and *Lgmn* mRNA expression (*n* = 3). (B) Representative immunoblots of C/EBPβ, AEP, APP NT, APP N585, Tau5, Tau N368, and phosphorylated Tau (p‐Tau Thr205 and p‐Tau S396) in the hippocampus. (C) Densitometric quantification of immunoblots shown in (B) (*n* = 4). (D) ELISA quantification of hippocampal Aβ40 and Aβ42 levels (*n* = 8). Data are presented as mean ± SEM. Normality was assessed using the Shapiro–Wilk test, and variance homogeneity was tested by Bartlett's test. Statistical significance was assessed using one‐way ANOVA with Dunnett's post hoc test (A, C) or Dunnett T3 test for unequal variances (D). Statistical significance was determined as *p < 0.05; **p < 0.01; ***p < 0.001.

Given that Aβ deposition and tau hyperphosphorylation are key pathological hallmarks of AD, we examined whether BYHWD ameliorates these pathologies via modulation of the C/EBPβ/AEP signaling pathway. First, we analyzed the expression levels of *Cebpb* and *Lgmn* mRNA in the hippocampus by qPCR (Figure 2A). Compared with the control group, the model group exhibited marked upregulation of both genes. Treatment with BYHWD‐L or BYHWD‐H significantly reduced the expression of *Cebpb* and *Lgmn*, indicating transcriptional suppression of this pathway in the hippocampus of 3×Tg mice.

Subsequently, we examined the protein expression of key components in the C/EBPβ/AEP pathway using Western blotting (Figure 2B,C). Compared to the Control group, the Model group exhibited markedly elevated levels of C/EBPβ, AEP, APP NT, APP N585, Tau 5, Tau N368, and phosphorylated Tau (p‐Tau Thr205 and p‐Tau S396), indicating pronounced activation of this pathological cascade. Treatment with BYHWD‐H significantly suppressed the expression of these proteins, bringing most targets close to baseline levels observed in the control group. BYHWD‐L also exerted a moderate inhibitory effect, though to a lesser extent than the high‐dose group. Similarly, donepezil treatment attenuated the upregulation of C/EBPβ and its downstream effectors.

#### 3.1.3 BYHWD Reduces Aβ Deposition and Tau Hyperphosphorylation, Ameliorating Neurodegeneration in 3×Tg Mice

To evaluate the effect of BYHWD on Aβ plaque deposition, we quantified hippocampal levels of the two major Aβ isoforms, Aβ40 and Aβ42, using ELISA. Both peptides were markedly elevated in the 3×Tg model mice compared to controls (Figure 2D), consistent with AD pathology. Treatment with BYHWD‐L partially reduced Aβ40 and Aβ42 accumulation, while BYHWD‐H and donepezil produced more robust reductions, restoring peptide levels closer to those in the control group. These findings indicate that BYHWD effectively attenuates Aβ overproduction in the hippocampus of 3×Tg mice.

**FIGURE 3:**
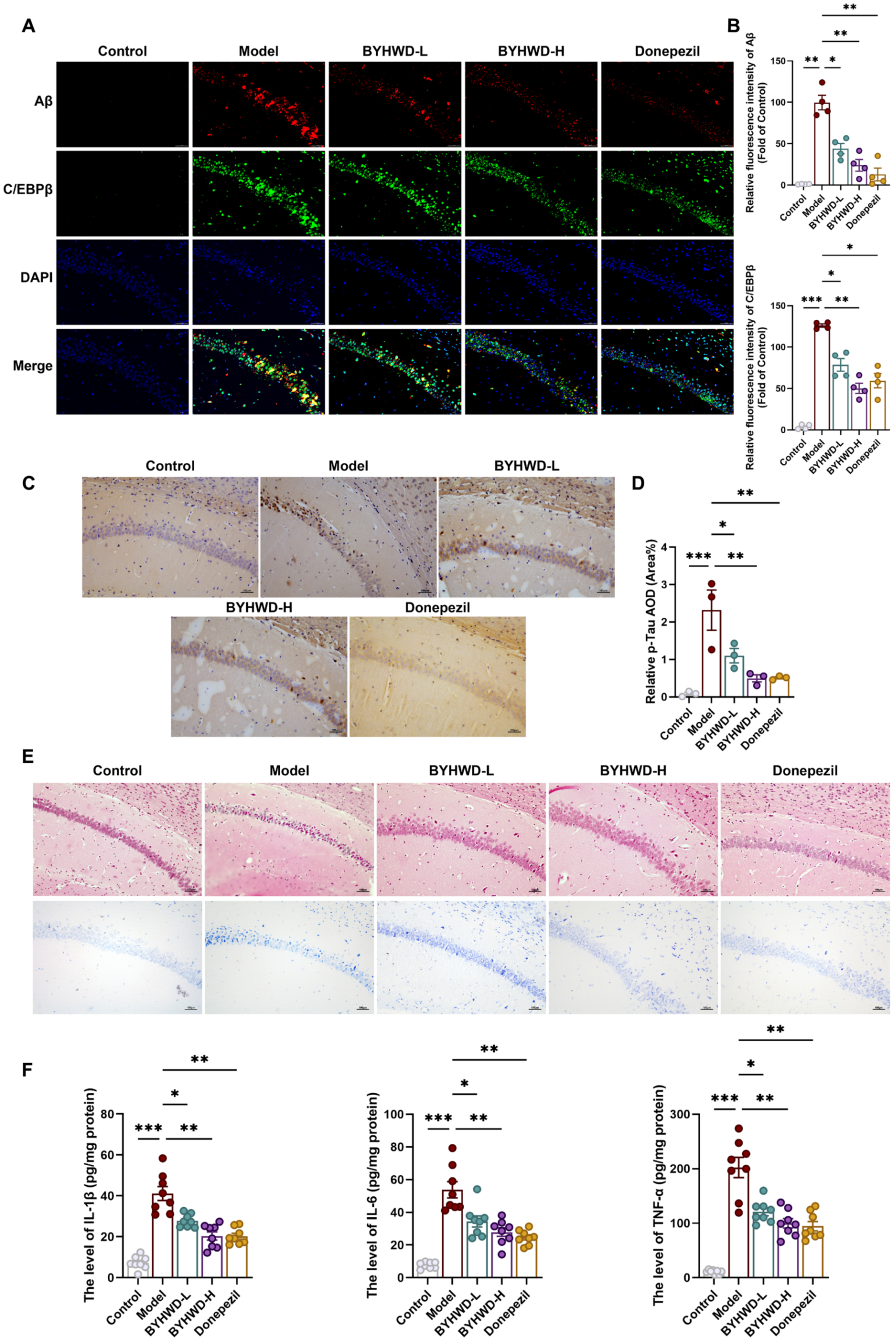
BYHWD attenuates C/EBPβ‐mediated Aβ and Tau pathology and reduces neuroinflammation in 3×Tg mice. (A) Immunofluorescence staining of Aβ (red) and C/EBPβ (green) in hippocampal sections (200×, scale bar = 50 μm). (B) Quantification of Aβ and C/EBPβ fluorescence intensity (*n* = 4). (C) Immunohistochemical detection of p‐Tau in the hippocampus (200×, scale bar = 100 μm). (D) Quantification of p‐Tau expression from (C) (*n* = 3). (E) H&E and Nissl staining showing histopathological alterations in the CA1 region (200×, scale bar = 100 μm). (F) ELISA quantification of hippocampal IL‐1β, IL‐6, and TNF‐α levels (*n* = 8). Data are presented as mean ± SEM. Normality was assessed using the Shapiro–Wilk test, and variance homogeneity was tested by Bartlett's test. Statistical significance was assessed using one‐way ANOVA with Dunnett's post hoc test (B) or Dunnett T3 test for unequal variances (D, F). Statistical significance was determined as *p < 0.05; **p < 0.01; ***p < 0.001.

Immunofluorescence analysis further revealed pronounced C/EBPβ overexpression and extensive Aβ plaque deposition in the hippocampi of model mice (Figure 3A,B). Both low‐ and high‐dose BYHWD treatments significantly suppressed C/EBPβ expression and concomitantly reduced Aβ plaque burden. A similar pattern was observed with donepezil, suggesting that the reduction in Aβ pathology is mechanistically linked to the downregulation of C/EBPβ.

In parallel, immunohistochemical staining showed marked elevation of p‐Tau in the hippocampus of 3×Tg mice (Figure 3C,D). Treatment with BYHWD, particularly at the higher dose, substantially decreased p‐Tau accumulation, comparable to the effect of donepezil. This suggests that BYHWD mitigates tau hyperphosphorylation, a critical driver of neurofibrillary tangle formation and neuronal dysfunction in AD.

To examine structural neuronal integrity, we performed HE and Nissl staining in the hippocampal CA1 region (Figure 3E). Control mice displayed well‐organized pyramidal neurons with prominent, intact Nissl bodies, whereas 3×Tg mice exhibited neuronal loss, irregular arrangement, and diminished Nissl substance. BYHWD‐treated groups, especially at high dose, showed considerable improvements in neuronal morphology, density, and Nissl body restoration, indicative of neuroprotective effects.

#### 3.1.4 BYHWD Attenuates Hippocampal Neuroinflammation in 3×Tg Mice

Given the central role of C/EBPβ in orchestrating neuroinflammatory responses during AD progression, we next investigated whether BYHWD modulates hippocampal inflammation in 3×Tg mice. ELISA analysis revealed markedly elevated levels of pro‐inflammatory cytokines—IL‐1β, IL‐6, and TNF‐α—in the hippocampus of model mice compared to controls (Figure 3F). Treatment with BYHWD‐L, BYHWD‐H, and donepezil significantly reduced the expression of these cytokines, with the high‐dose BYHWD and donepezil showing more pronounced effects. These results highlight the anti‐inflammatory efficacy of BYHWD in attenuating neuroinflammation in the AD brain.

Collectively, these results suggest that BYHWD exerts significant protective effects on the neurons of 3×Tg mice, reducing pathological Aβ deposition, tau hyperphosphorylation, and the release of pro‐inflammatory cytokines. The underlying mechanism appears to be associated with the suppression of the C/EBPβ/AEP signaling pathway by BYHWD.

### 3.2 BYHWD Restores Gut Microbial Dysbiosis in 3×Tg Mice

α‐diversity analysis (Figure 6A) revealed that the gut microbiota richness and diversity in the Model group were significantly diminished compared to the Control group. Specifically, in 3×Tg mice, the Chao1, Observed species, Simpson, and Shannon indices were all significantly reduced, indicating gut microbial dysbiosis. In contrast, treatment with BYHWD‐L and BYHWD‐H significantly enhanced microbial richness and diversity, with the most prominent effects observed in the BYHWD‐H group. These results suggest that BYHWD, especially at the high dose, effectively restores gut microbiota diversity and richness in 3×Tg mice.

The ASV/OTU Venn diagram (Figure 6B) further demonstrated a significant reduction in the number of ASVs/OTUs in the Model group compared to the Control group. However, after treatment with BYHWD‐L, BYHWD‐H, and Donepezil, the number of ASVs/OTUs in these groups increased, approaching the levels observed in the Control group. To assess microbial community composition differences between groups, β‐diversity analysis was performed using PCoA (Weighted UniFrac) and NMDS visualizations. As shown in Figure 6C, both PCoA and NMDS analysis revealed a distinct separation in microbial community composition between the Control and Model groups. After BYHWD‐L and BYHWD‐H treatments, microbial communities exhibited clustering patterns that progressively shifted toward the Control group, with an increased divergence from the Model group.

#### 3.2.1 BYHWD Modulates Gut Microbiota Composition at the Phylum Level in 3×Tg Mice

To analyze gut microbial composition at different taxonomic levels, we conducted a systematic evaluation of fecal samples. As shown in Figure 6D, at the phylum level, the relative abundance of *Bacteroidota* in the Model group increased compared to the Control group, while the proportion of *Firmicutes* decreased. Additionally, the abundance of *Proteobacteria* was significantly elevated in the Model group compared to the Control and BYHWD‐H groups. Treatment with BYHWD‐L and BYHWD‐H significantly restored the relative abundance of *Firmicutes* and *Bacteroidota*, indicating a modulatory effect of BYHWD on gut microbiota composition.

#### 3.2.2 BYHWD Modulates Gut Microbiota Composition at the Family Level in 3×Tg Mice

At the family level (Figure 6E), the relative abundance of *Staphylococcaceae* was significantly increased in the Model group, while *Oscillospiraceae* levels were significantly reduced. Treatment with both BYHWD‐L and BYHWD‐H effectively reversed these changes, restoring the relative abundance of *Staphylococcaceae* and *Oscillospiraceae* to levels comparable to the Control group.

#### 3.2.3 BYHWD Modulates Gut Microbiota Composition at the Genus Level in 3×Tg Mice

At the genus level (Figure 6F), the relative abundance of *Atopostipes* was markedly elevated in the Model group, while *Prevotella* sp. and *Duncaniella* were significantly reduced. Treatment with BYHWD‐L and BYHWD‐H led to a significant reduction in *Atopostipes* abundance and a notable increase in *Lachnospiraceae* and *Prevotella* sp. levels. Furthermore, BYHWD‐L treatment also significantly elevated the abundance of *Duncaniella*.

#### 3.2.4 Differential Gut Microbiota Analysis Through LefSe in BYHWD‐Treated 3×Tg Mice

LefSe analysis was performed to identify species with significant abundance differences between groups (Figure 6G,H). Following BYHWD treatment, we observed a significant enrichment of *Muribaculaceae* and *Paramuribaculum* in the treated groups. In contrast, in the Model group, significantly enriched taxa included *Firmicutes_D*, *Bacilli*, *Staphylococcales*, *Bacillales_A*, *Bacillales_B*, *Bacillales_D*, *Actinomycetales*, *Mammaliicoccus*, *Carnobacteriaceae*, *Planococcaceae*, *Sporosarcina*, *Aerococcaceae*, *Facklamia_A*, *Salinicoccaceae*, *Jeotgalicoccus_A*, *Amphibacillaceae*, *Bacillaceae_C*, and *Lederbergia*.

### 3.3 FMT‐BYHWD Ameliorates Behavioral Impairments, AD‐Like Pathology, and the C/EBPβ/AEP Signaling Pathway in 3×Tg Mice

**FIGURE 4:**
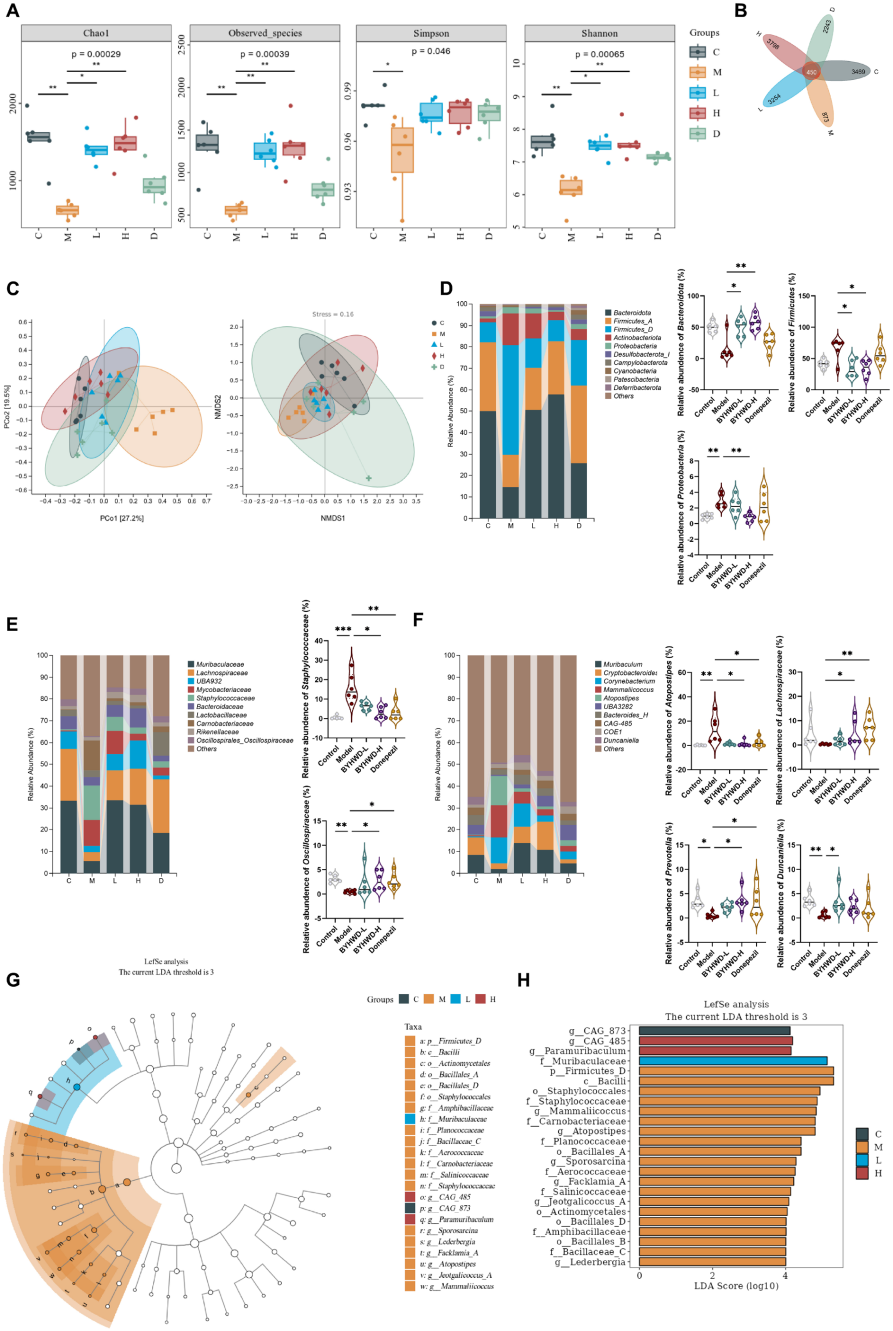
BYHWD restores gut microbiota diversity and composition in 3×Tg mice. (A) Alpha diversity indices (Chao1, Observed species, Shannon, Simpson) indicating microbial richness and diversity across groups (*n* = 6). (B) Venn diagram displaying shared and unique OTUs across groups. (C) Principal coordinates analysis (PCoA) and non‐metric multidimensional scaling (NMDS) revealing compositional differences in microbial communities. (D–F) Relative abundance of gut microbiota at the phylum (D), family (E), and genus (F) levels. (G, H) Linear discriminant analysis (LDA) identifying taxa differentially enriched between groups. Data are presented as median with interquartile range (IQR) for violin plots and box plots. Diversity indices (Chao1, Observed species, Shannon, Simpson) were calculated, and statistical differences were assessed using the Kruskal–Wallis test with Dunn's post hoc test for pairwise comparisons (A). Beta diversity was assessed using the UniFrac distance metric, with visualization by PCoA and NMDS (C). Normality was tested using the Shapiro–Wilk test, followed by one‐way ANOVA for normally distributed data and the Kruskal–Wallis test for non‐normally distributed data (D–F). Taxonomic composition at the phylum, family, and genus levels was analyzed and visualized using QIIME2‐generated histograms (D–F). *p < 0.05; **p < 0.01; ***p < 0.001.

**FIGURE 5:**
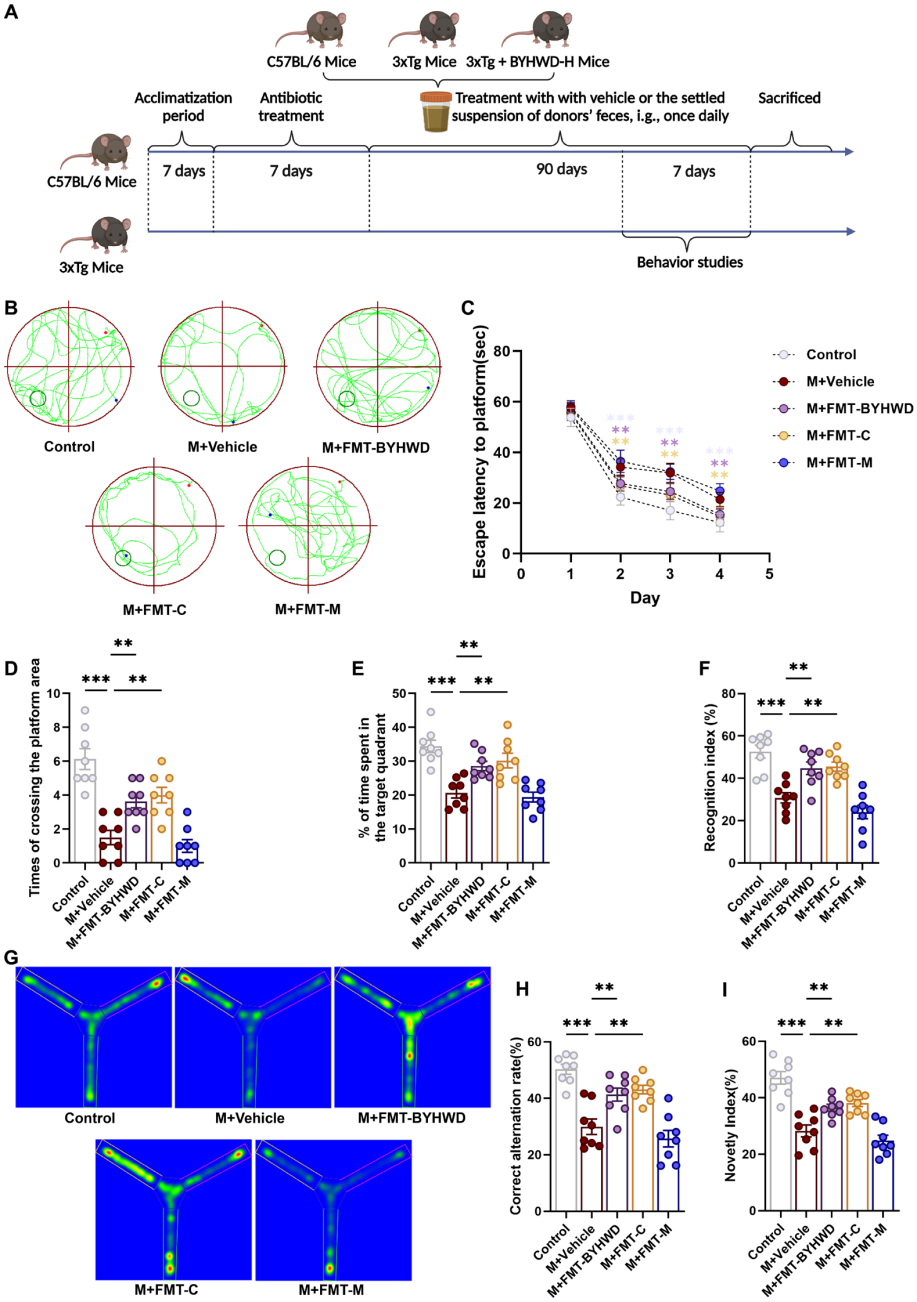
FMT‐BYHWD ameliorates behavioral and cognitive impairments in 3×Tg mice. (A) Experimental timeline and schematic illustrating the second phase of the study. (B) Representative swimming trajectories from the Morris water maze probe trial. (C) Escape latency across the four training days (Days 1–4) in the Morris water maze test, reflecting spatial memory acquisition. Data are shown as mean ± SD (*n* = 8). (D) Number of platform crossings during the Morris water maze probe trial, indicating spatial memory retention. (E) Time spent in the target quadrant during the Morris water maze probe trial, assessing memory performance. (F) Recognition index calculated from exploration time of the novel versus familiar object in the novel object recognition test. (G) Representative heatmaps showing exploration behavior in the Y‐maze, reflecting preference for the novel arm as an indicator of working memory and cognitive flexibility. (H) Percentage of correct alternations in the Y‐maze, measuring working memory and cognitive flexibility. (I) Novelty index in the Y‐maze, quantifying preference for the novel arm as an indicator of exploratory behavior and memory. Data are presented as mean ± SEM (*n* = 8) unless otherwise specified. Normality was assessed using the Shapiro–Wilk test, and variance homogeneity was tested by Bartlett's test. Statistical significance was assessed using one‐way ANOVA with Dunnett's post hoc test (D–F, H, I) and two‐way ANOVA with Bonferroni's post hoc test (C). Statistical significance was determined as *p < 0.05; **p < 0.01; ***p < 0.001.

Previous studies have demonstrated that BYHWD restores gut microbial dysbiosis and inhibits the activation of the C/EBPβ/AEP signaling pathway (Figures 2, 3, 4). To further determine whether BYHWD exerts its anti‐AD effects by modulating the gut microbiota and subsequently suppressing the C/EBPβ/AEP pathway, we administered FMT‐BYHWD treatment to 3×Tg mice (Figure 5A).

#### 3.3.1 FMT‐BYHWD Ameliorate Learning and Memory Deficits in 3×Tg AD Mice

The Morris water maze test (Figure 5C) revealed that both the M + Vehicle and M + FMT‐M groups had longer escape latencies from Day 2 to Day 4 compared to the Control group. In contrast, both the M + FMT‐BYHWD and M + FMT‐C groups showed significant reductions in escape latency, indicating improved learning. Over the training period, all groups demonstrated a gradual decline in escape latency, reflecting enhanced learning performance. In the spatial probe test (Figure 5B–E), the M + Vehicle and M + FMT‐M groups spent less time in the target quadrant and made fewer platform crossings compared to the Control group. However, the M + FMT‐BYHWD and M + FMT‐C groups showed increased platform crossings and more time spent in the target quadrant, suggesting improved memory retention.

The novel object recognition test (Figure 5F) showed impaired recognition memory in the M + Vehicle and M + FMT‐M groups, as indicated by a lower recognition index compared to the Control group. In contrast, both the M + FMT‐BYHWD and M + FMT‐C groups exhibited significantly better object recognition.

Y‐maze analysis (Figure 5G–I) revealed a reduced spontaneous alternation rate and novelty preference in the M + Vehicle and M + FMT‐M groups compared to the Control group. Both the M + FMT‐BYHWD and M + FMT‐C groups demonstrated enhanced spontaneous alternation and novelty preference, indicative of improved cognitive flexibility.

These findings collectively suggest that FMT‐BYHWD and FMT‐C treatments significantly ameliorate learning and memory deficits in 3×Tg mice, highlighting the potential therapeutic benefits of BYHWD in neurodegenerative diseases through modulation of gut microbiota.

#### 3.3.2 FMT‐BYHWD Inhibits the Overactivated C/EBPβ/AEP Signaling Pathway in 3×Tg Mice

Experiment 1 result suggested that BYHWD modulates gut microbiota to regulate the C/EBPβ/AEP signaling pathway, contributing to the attenuation of AD pathology. To further explore this mechanism, we examined the impact of FMT‐BYHWD on key molecular markers involved in AD pathology.

**FIGURE 6:**
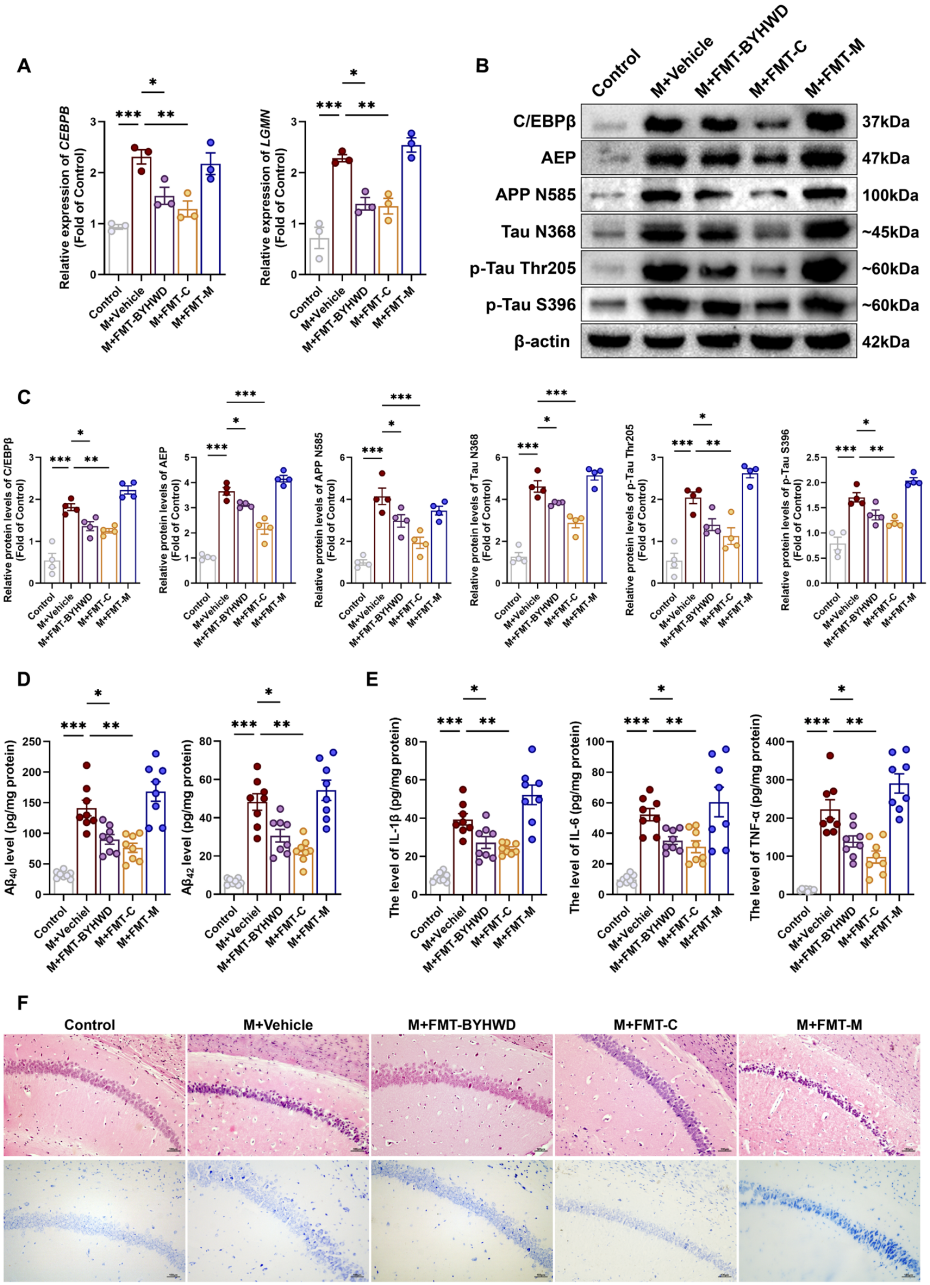
FMT‐BYHWD suppresses the C/EBPβ‐AEP pathway, attenuates Aβ and Tau pathology, and reduces neuroinflammation in 3×Tg mice. (A) qPCR analysis of hippocampal *Cebpb* and *Lgmn* mRNA expression (*n* = 3). (B) Representative immunoblots of C/EBPβ, AEP, APP N585, Tau N368, and phosphorylated Tau (p‐Tau Thr205 and p‐Tau S396) in the hippocampus. (C) Densitometric quantification of immunoblots shown in (B) (*n* = 4). (D) ELISA quantification of hippocampal Aβ40 and Aβ42 levels (*n* = 8). (E) ELISA analysis of hippocampal IL‐1β, IL‐6, and TNF‐α levels (*n* = 8). (F) Representative images of HE and Nissl staining showing histopathological alterations in the CA1 region of the hippocampus (200×, scale bar = 100 μm). Data are presented as mean ± SEM. Normality was assessed using the Shapiro–Wilk test, and variance homogeneity was tested by Bartlett's test. Statistical significance was assessed using one‐way ANOVA with Dunnett's post hoc test (A, C) or Dunnett T3 test for unequal variances (D, E). Statistical significance was determined as *p < 0.05; **p < 0.01; ***p < 0.001.

We assessed the impact of FMT‐BYHWD on the expression of *Cebpb* and *Lgmn* mRNA in the hippocampus of 3×Tg mice. As shown in Figure 6A, mRNA levels of *Cebpb* and *Lgmn* were elevated in the M + Vehicle and M + FMT‐M groups relative to the Control group. In contrast, FMT‐BYHWD and FMT‐C treatments significantly reduced the expression of both genes, indicating that FMT‐BYHWD inhibits the upregulation of *Cebpb* and *Lgmn* in the hippocampus.

In further investigations of protein expression, we examined the effect of FMT‐BYHWD on the protein expression of C/EBPβ, AEP, APP N585, Tau N368, p‐Tau Thr205, and p‐Tau S396. As shown in Figure 6B,C, expression of C/EBPβ, AEP, APP N585, p‐Tau Thr205, and p‐Tau S396 was significantly higher in the M + Vehicle and M + FMT‐M groups compared to the Control group. FMT‐BYHWD and FMT‐C treatment led to a marked reduction in the expression of these proteins, suggesting that the gut microbiota in FMT‐BYHWD‐treated 3×Tg mice attenuates C/EBPβ/AEP signaling and mitigates Tau hyperphosphorylation.

Together, these findings indicate that FMT‐BYHWD modulates gut microbiota to mitigate the overactivation of the C/EBPβ/AEP signaling pathway and reduce Tau phosphorylation, thus highlighting its potential as a therapeutic strategy for AD through gut–brain axis modulation.

## 4 Discussion

This study provides the first evidence that BYHWD modulates the gut microbiome–C/EBPβ/AEP signaling axis, restoring gut microbiome balance and downregulating C/EBPβ expression in the hippocampus. Inhibition of C/EBPβ activity reduced AEP‐mediated cleavage of APP and Tau hyperphosphorylation, further underscoring the neuroprotective effects of BYHWD in the AD model and offering novel mechanistic insights for AD intervention. Moreover, this study extends the potential therapeutic applications of the gut–brain axis and the C/EBPβ/AEP pathway in the pathogenesis and treatment of AD.

Behavioral assessments in 3×Tg mice revealed significant cognitive deficits, including reduced platform crossing, decreased time spent in the target quadrant, impaired novel object recognition, reduced alternation, and diminished spatial exploration—key features of AD‐related cognitive impairment [21, 22, 23, 24, 25]. Treatment with BYHWD significantly ameliorated these behavioral deficits, further supporting its protective effects on cognitive function.

The key pathological features of AD include Aβ deposition and Tau hyperphosphorylation [26, 27, 28, 29, 30]. Aβ oligomers and plaques trigger pathological responses in microglia, astrocytes, and neurons, contributing to neuronal loss [26, 27, 31]. Specifically, Tau hyperphosphorylation at Thr205, S422, and S396 correlates with disease progression [32, 33, 34, 35, 36, 37, 38]. The C/EBPβ/AEP signaling axis has been recognized as a critical pathway in AD pathogenesis [12]. Overexpression of C/EBPβ enhances AEP activation, resulting in abnormal cleavage of APP and Tau, which exacerbates Aβ accumulation and Tau hyperphosphorylation [39, 40]. Inhibition of this pathway alleviates AD‐associated pathological changes. In line with this, transgenic mice expressing C/EBPβ show reduced Aβ and Tau pathology when the axis is suppressed [41, 42]. Similarly, overexpression of C/EBPβ in the hippocampus of TgCRND8 mice increases AEP activity, leading to the enhanced generation of APP N585 and Tau N368 fragments, which promotes Tau phosphorylation and Aβ accumulation [43]. In the present study, we further confirm the aberrant activation of the C/EBPβ‐AEP axis in the hippocampus of 3×Tg mice and demonstrate for the first time that BYHWD inhibits this pathway, reducing the generation of APP N585 and Tau N368 fragments, significantly lowering Aβ40 and Aβ42 levels, and suppressing Tau hyperphosphorylation at Thr205 and Ser396. Immunofluorescence analysis revealed a significant decrease in C/EBPβ expression and a reduction in Aβ load following BYHWD treatment, indicating that BYHWD exerts neuroprotective effects by modulating the C/EBPβ‐AEP axis to attenuate Aβ and Tau‐related pathological changes.

Neuroinflammation is a central driver of AD progression, with Aβ deposition activating microglia and astrocytes, leading to the release of pro‐inflammatory cytokines such as IL‐1β, IL‐6, and TNF‐α [44, 45, 46, 47, 48, 49]. This cascade exacerbates tau phosphorylation and Aβ aggregation. The aberrant activation of the C/EBPβ‐AEP axis, resulting in a significant increase in inflammatory cytokines, is closely associated with neuroinflammation in AD [50, 51, 52]. In this study, we demonstrate that treatment with BYHWD significantly reduced the levels of pro‐inflammatory cytokines in the hippocampus of 3×Tg mice, suggesting that BYHWD may attenuate neuroinflammation and slow the neurodegenerative processes in AD by inhibiting the C/EBPβ‐AEP axis.

The involvement of gut microbiota in clinical AD patients and experimental AD models is well‐documented, with dysbiosis influencing disease progression through various mechanisms [5, 53, 54]. Building upon this, the present study further investigates the interaction between gut microbiota and the C/EBPβ‐AEP axis in AD. Dysbiosis in 5xFAD mice has been shown to intensify the activation of the C/EBPβ‐AEP axis, accelerating neurodegeneration [55]. Additionally, research using Thy1‐C/EBPβ transgenic mice reveals that transplanting microbiota from AD patients induces AD‐like pathological features in the transgenic mice. This suggests that specific microbial communities, such as *Bacteroides fragilis* and its associated metabolites, may influence the development of AD [56]. This “gut–brain” axis positions gut microbiota as a potential target for modulating key pathways involved in AD pathology.

Regulation of the gut microbiota has emerged as a promising therapeutic strategy for neurodegenerative diseases. In this study, we demonstrate that BYHWD significantly reshapes the gut microbiota composition in 3×Tg mice, reversing AD‐associated dysbiosis. At the phylum, family, and genus levels, BYHWD effectively restores the balance between *Firmicutes/Bacteroidetes* (F/B) ratio and significantly reduces the abnormal increase in *Staphylococcaceae*, a microbiota genus closely linked to neuroinflammation [57, 58, 59]. The phylum *Bacteroidetes* has been shown to inhibit microglial clearance of Aβ, promoting the deposition of Aβ plaques, which correlates with the increase in hippocampal Aβ accumulation observed in this study [58]. Additionally, BYHWD modulated the family‐level abundance of *Staphylococcus*, a pro‐inflammatory genus implicated in neuroinflammation and AD pathogenesis [60, 61]. At the genus level, BYHWD treatment resulted in a significant reduction of *Prevotella* sp. and an upregulation of *Atopostipes*, which may contribute to restoring gut barrier integrity, thereby alleviating neuroinflammation and AD‐associated pathological changes [62, 63]. These findings suggest that BYHWD may exert neuroprotective effects through gut microbiota regulation.

Fecal microbiota transplantation (FMT) is one of the most effective interventions for modulating the gut microbiota. The mechanism involves introducing fecal matter from a healthy donor into the gastrointestinal tract of a recipient, thereby rebalancing the gut microbiota and achieving therapeutic effects. Recent studies have highlighted the therapeutic potential of FMT across various diseases, including autism spectrum disorders, optic neuritis, stroke, multiple system atrophy, and neurodegenerative diseases like AD [64, 65]. Following FMT, the recipient's gut microbiota typically restores to a state similar to that of the healthy donor [66, 67, 68, 69]. For example, it has been shown that transplanting a healthy microbiota into germ‐free AD transgenic mouse models improves Aβ and tau pathology [70]. Similar results were observed in another AD mouse model, where transplanting microbiota from wild‐type mice into germ‐free APP/PS1 transgenic mice alleviated Aβ pathology in the brain, while transplanting microbiota from conventionally housed APP/PS1 mice exacerbated Aβ accumulation [71].

We further validate the anti‐AD effects of BYHWD through gut microbiota modulation. FMT combined with BYHWD treatment significantly improved cognitive function in 3×Tg mice. Additionally, inhibition of the C/EBPβ/AEP signaling pathway enhanced therapeutic efficacy, suggesting that BYHWD may exert neuroprotective effects by regulating the C/EBPβ‐AEP axis via gut microbiota modulation. Transplantation of healthy donor microbiota into AD mice reduced Aβ deposition and tau pathology, while microbiota from AD mice exacerbated these changes, highlighting the critical role of gut microbiota in AD pathogenesis [70]. Interestingly, in 7‐month‐old 3×Tg mice, FMT treatment did not worsen cognitive decline or AD‐like pathological changes. No significant differences were observed between FMT‐M‐treated mice and drug‐treated controls in behavioral performance or AD‐related molecular mechanisms. This may be due to age‐related exacerbation of gut dysbiosis, insufficient aging of the donor microbiota, or the relatively short duration of FMT treatment [72, 73, 74]. These findings suggest that the microbiota's impact on AD progression may be time‐sensitive, emphasizing the need for further investigation into the role of age in FMT‐based interventions.

Despite the progress made, several limitations must be considered. Although BYHWD significantly alters the gut microbiota composition in 3×Tg mice, the causal relationship between specific microbial populations and its neuroprotective effects remains unclear. Mechanistic validation in germ‐free or gnotobiotic models is needed to confirm direct microbial contributions to AD pathology [75]. Furthermore, identifying the bioactive components of BYHWD and their molecular targets in the gut–brain axis is critical for elucidating its therapeutic mechanisms. While this study highlights the key microbial populations regulated by BYHWD, further research is required to determine the signaling pathways through which these microbes influence neurodegeneration. Additionally, the long‐term effects of BYHWD on AD progression and its clinical relevance warrant further investigation. Future studies should integrate multi‐center clinical trials with humanized organoid and iPSC‐based models, which will be crucial for translating these findings into effective, personalized therapies.

From a translational medicine perspective, this study provides new experimental evidence supporting microbiota modulation targeting the C/EBPβ‐AEP axis, with significant clinical application potential. Microecological interventions, particularly those involving TCM (such as BYHWD), probiotics, FMT, or specific microbial metabolites, may offer novel therapeutic approaches for AD. By modulating the gut microbiota and targeting the C/EBPβ‐AEP axis, neuroinflammation can be alleviated, neuronal function improved, and the onset and progression of AD potentially delayed. In the future, integrating multi‐omics approaches, including metagenomics, metabolomics, and single‐cell transcriptomics, will provide comprehensive insights into these complex interactions, helping to advance microbiota‐targeted therapeutic strategies. Therefore, it is crucial to validate these findings in humanized models and clinical cohorts to bring microbiota‐based AD interventions closer to clinical application.

## 5 Conclusion

In conclusion, this study provides robust evidence supporting the therapeutic potential of the TCM formula BYHWD in ameliorating cognitive deficits in 3×Tg mice. Our findings suggest that BYHWD exerts its effects by modulating the gut microbiota–C/EBPβ/AEP axis, which in turn mitigates Aβ plaque deposition, Tau hyperphosphorylation, and neuroinflammatory responses. Given the complex pathophysiology of AD, conventional strategies focusing on single‐pathway inhibition have often yielded limited success. By contrast, our findings highlight the gut microbiota as an emerging therapeutic target, suggesting that BYHWD‐mediated microbiome modulation represents a novel, non‐invasive, and multifaceted approach to delaying AD onset and progression. This innovative strategy offers new insights into microbiota‐based interventions for neurodegenerative diseases. Future investigations should focus on identifying the key bioactive components of BYHWD, elucidating their specific microbiota‐mediated actions, and exploring their translational potential in clinical applications. These findings lay the groundwork for the development of microbiome‐based therapeutic strategies in AD, bridging traditional medicine with modern precision medicine approaches.

## Author Contributions

J.L. and X.D. contributed to the original draft preparation, validation, software, methodology, and investigation. J.L., J.Y., and N.H. were responsible for data collection and integration. B.L., X.L., and J.C. contributed to conceptualization. B.L., J.C., and S.C. supervised the project. B.L. and S.C. administered the project. X.D., J.C., and W.Z. acquired funding. All authors reviewed and approved the final manuscript.

## Conflicts of Interest

The authors declare no conflicts of interest.

## Supporting information


Methods S1.

## Data Availability

The data supporting the findings of this study are available from the corresponding author, Bin Liu, upon reasonable request.
