# Supplementary material for: Buyang Huanwu Decoction Modulates the Gut Microbiota–C/EBPβ/AEP Axis to Ameliorate Cognitive Impairment in Alzheimer's Disease Mice
Source: CNS Neurosci Ther. 2025 Jun 23;31(6):e70480. doi: 10.1111/cns.70480 (PMC12183522; doi:10.1111/cns.70480)
Supplement: Supplementary file 1 — Methods S1. [file CNS-31-e70480-s001.docx]

Methods

Animals

A total of 64 SPF-grade male 3xTg mice (2-3 months old, 15-20g) were procured from Beijing Huafukang Bioscience Co., Ltd. (Beijing, China), under certificate number SCXK (Jing) 2024-0003. Additionally, 16 SPF-grade male C57BL/6 mice (2-3 months old, 15-20g) were obtained from Liaoning Changsheng Biotechnology Co., Ltd. (Liaoning, China), under certificate number SCXK (Liao) 2020-0001. The mice were housed in a temperature-controlled environment at 22±1°C with a relative humidity of 60±10%. They were maintained on a 12-hour light/dark cycle and provided with ad libitum access to food and water, with standardized feeding conditions. All animal experiments were conducted following the ethical guidelines outlined in the 3Rs principles of animal experimentation, ensuring minimal harm and distress to the animals. The study protocols were approved by the Animal Ethics Committee of Heilongjiang University of Chinese Medicine (Ethics approval number: 12352135).

### **Preparation of BYHWD**

BYHWD is composed of 120g *Astragalus mongholicus Bunge*, 6g *Angelica sinensis* (Oliv.) Diels, 4.5g *Paeonia veitchii Lynch*, 3g *Ligusticum chuanxiong*, 3g *Prunus persica* (L.) Batsch, 3g *Carthamus tinctorius* L., and 3g *Pheretima aspergillum* (E.Perrier), combined in a ratio of 120:6:4.5:3:3:3:3 (Table.1). All crude herbal materials were sourced from Beijing Tongrentang (Harbin branch, Institution Code: P23990000014). A total of 1425 g of raw herbs were ground into coarse powder, soaked in distilled water for 2 hours, and decocted for 2 hours using a large-scale rotary evaporator. This extraction process was repeated three times, each for 1 hour. The combined filtrates were concentrated under reduced pressure and subsequently freeze-dried to yield 486.07 g of dry powder, corresponding to a yield of 34.11% (i.e., 1 g of dry extract equals 2.93 g of raw materials). The resulting lyophilized powder was stored in a sealed, desiccated container at 4 °C until use. Prior to administration, the powder was reconstituted in distilled water, thoroughly mixed, and heated in a water bath.

**Table 1. Composition of Buyang Huanwu Decoction(BYHWD)**

| Traditional Chinese Medicine | Botanical name | Part Used | Dosage used | Place of Origin | Batch Number |
| --- | --- | --- | --- | --- | --- |
| Huang Qi | *Astragalus mongholicus Bunge* | Root | 120g | Gansu | 20230801 |
| Dang Gui Wei | *Angelica sinensis* (Oliv.) Diels | Root | 6g | Gansu | 20220526 |
| Chi Shao | *Paeonia veitchii Lynch* | Root | 4.5g | Inner Mongolia | C168240301 |
| Chuan Xiong | *Ligusticum chuanxiong* | Root | 3g | Sichuan | 20220501 |
| Tao Ren | *Prunus persica* (L.) Batsch | Seed | 3g | \| Hebei \| \| --- \| | 20220801 |
| Hong Hua | *Carthamus tinctorius* L. | Flower | 3g | Xinjiang | 20220801 |
| Di Long | *Pheretima aspergillum* (E.Perrier) | Whole | 3g | Guangxi | 240101 |

The plant names have been checked with “The World Flora Online” (http://www.worldfloraonline.org) (accessed on April 24, 2025)

Animal Grouping and Administration

Experiment 1: To investigate the effects of BYHWD on 3xTg mice, 2–3-month-old male 3xTg mice were randomly assigned to four groups (n = 8): Model, BYHWD-L (9.26 g/kg/day), BYHWD-H (37.05 g/kg/day), and Donepezil (5 mg/kg/day). Age-matched male C57BL/6 mice were used as the Control group (n = 8). BYHWD lyophilized powder was dissolved in distilled water and administered via oral gavage. Donepezil hydrochloride (PHR1584-1G; Macklin, Shanghai, China) was prepared similarly. Mice in the Control and Model groups received equivalent volumes of distilled water. Treatments were administered daily for 90 consecutive days. At the end of the treatment period, mice were subjected to behavioral tests, including the Morris water maze test (MWMT), novel object recognition test (NORT), and Y-maze test. Mice were euthanized under deep anaesthesia, and brain tissues were collected for subsequent analysis. The experimental timeline is shown in Fig. 1A.

The BYHWD dosing regimen was based on the standard clinical prescription dose of 142.5 g/day. Assuming an average adult body weight of 60 kg, the human dose equals ~2.04 g/kg/day. Using the recommended human-to-mouse dose conversion based on body surface area, the equivalent mouse dose is ~29.2 g/kg/day ^1,2^. Therefore, we selected a low dose (9.26 g/kg/day), representing approximately one-third of the equivalent dose, and a high dose (37.05 g/kg/day), which slightly exceeds the equivalent dose, to assess potential dose-response relationships ^2^. This dosing strategy is consistent with previous studies reporting efficacy and safety of BYHWD in neurodegenerative disease models ^3-5^.

Experiment 2: To evaluate the role of gut microbiota in the therapeutic effects of BYHWD, fecal microbiota transplantation (FMT) was performed. Male 3xTg mice (2–3 months old) were randomly divided into four groups (n = 8): M+Vehicle, M+FMT-BYHWD, M+FMT-C, and M+FMT-M. Pseudo-germ-free mice were generated by gavaging 0.25 mL of an antibiotic cocktail once daily for 7 consecutive days. The cocktail consisted of neomycin (1.25 mg/ml, N6090F), ampicillin (2.5 mg/ml, A1178), and metronidazole (2.5 mg/ml, M0662F), all purchased from Biotopped (Beijing, China). On day 8, mice were treated with fecal suspensions (50 mg/ml in PBS) derived from 7-month-old donor mice in the Control, Model, or BYHWD-H groups from Experiment 1. Fecal material (0.6 ml per mouse) was administered once daily via oral gavage for 90 days. Behavioral tests were conducted at the end of treatment, followed by euthanasia and tissue collection under anaesthesia. The experimental timeline is shown in Fig. 5A.

Morris water maze test (MWMT)

Following previously established protocols ^6,7^, the MWMT was conducted to assess the spatial learning and memory capabilities of the mice. A circular water pool with a diameter of 120 cm and a height of 45 cm, filled with water at 24°C, was divided into four quadrants. The apparatus included a transparent platform, 10 cm in diameter, positioned at the center of the third quadrant, with its top submerged approximately 2.5 cm below the water surface. During the experiment, the mice were trained to locate the hidden platform over four consecutive days, with three trials per day. In each trial, the mice were gently placed in the water at varying starting points and given up to 60 seconds to find and climb onto the hidden platform. The time taken by the mice to reach the platform (escape latency) was recorded. If a mouse failed to locate the platform within 60 seconds, it was manually guided to the platform and allowed to remain there for 15 seconds, with the escape latency recorded as 60 seconds. On the fifth day, following the removal of the platform, a spatial probe test was performed, allowing the mice to swim freely for 60 seconds in each trial. The parameters of the mice’s performance were recorded using the SuperMaze video tracking system.

Novel object recognition (NORT)

Prior to commencing the formal experiment, animals underwent adaptive training. During the experiment, the mice were placed in a 40 cm × 40 cm × 40 cm novel object recognition box, and their activity over a 5-minute period, including the total distance traveled, was recorded. On Day 1, the mice were allowed to freely explore the arena for 10 minutes (with no objects present, data from this session were used for open-field analysis) to acclimate to the environment. On Day 2, two identical objects (red cylindrical columns with a diameter of 2.5 cm and a height of 6 cm, defined as Object A) were placed in the arena, and the mice were allowed to explore for 10 minutes. On Day 3, one of the Objects A was replaced with a novel object of a different color and shape (a green cube measuring 3×3×3 cm, defined as Object B). The adaptive phase of the experiment spanned three days. On the fourth day, two identical small objects were placed in the upper corners of the empty box, and the activity of the mice was recorded over a 5-minute period. One hour later, one of the small objects was replaced with a new one, and the preference of the mice for the novel object was observed over another 5-minute period. To minimize olfactory cues from previous subjects, all objects and equipment were cleaned with 70% ethanol prior to and after each test. The same experimental procedure was followed throughout. The recognition index was calculated as follows: Recognition Index = [(Exploration time of the novel object) / (Exploration time of the novel object + Exploration time of the familiar object)] × 100%.

Y-Maze Test

The Y-maze consists of three symmetrical, opaque arms, each measuring 30 cm in length, 8 cm in width, and 15 cm in height. The three arms were designated as the start arm, the novel arm, and the other arm, with the novel arm sealed off by a partition at the start of the experiment. Spontaneous Alternation Test: Initially, the mice were brought into the test room and acclimated for 2 hours. Subsequently, each mouse was placed at the starting point of the start arm and allowed to freely explore for 10 minutes. Software recorded the total number of entries and the number of alternations (an alternation being defined as sequential entry into three different arms). After each test, the Y-maze was cleaned, and 75% ethanol was used to eliminate any scent traces. The spontaneous alternation rate was calculated using the following formula: Spontaneous Alternation Rate = Number of alternations / (Total number of arm entries - 2) × 100%. Novel Arm Exploration Test: This test was divided into two phases. First, the mice were acclimated to the test environment for 2 hours, then allowed to freely explore the start arm and the other arm for 10 minutes. After a 2-hour interval, the mice were reintroduced to the Y-maze, the partition was removed, and the novel arm was opened, allowing the mice to freely explore for 5 minutes. The time spent in each arm was recorded. After each test, the Y-maze was cleaned and any residual scent removed using 75% ethanol. The novelty index was calculated as follows: Novelty Index = Time spent in the novel arm / (Total time spent in all three arms) × 100%.

Sample Collection

For tissue collection, Mice were deeply anesthetized with tribromoethanol (30 μL/g, intraperitoneal; MA0478, Meilunbio, Liaoning, China) prior to sacrifice. Cardiac perfusion was performed using ice-cold PBS to eliminate circulating blood. Brains from three mice per group were rapidly excised and fixed in 4% paraformaldehyde for histological analysis. For the remaining animals, hippocampal tissues were dissected, weighed, snap-frozen in liquid nitrogen, and stored at −80 °C for subsequent biochemical analyses, including ELISA, qPCR, and western blotting. Colonic fecal samples were collected under sterile conditions, flash-frozen in liquid nitrogen, and stored at −80 °C for 16S rRNA gene sequencing.

Pathological Staining

To examine histopathological alterations, brain tissues were collected following behavioral assessments and fixed in 4% paraformaldehyde for 24 h at 4 °C. Tissues were then dehydrated through a graded ethanol series, cleared in xylene, embedded in paraffin, and sectioned into 4-5 μm slices using a rotary microtome. Sections were subjected to hematoxylin and eosin (H&E) staining and Nissl staining according to the manufacturer’s instructions. Hematoxylin and Eosin Staining Kit (C0105S), Nissl Staining Solution (C0117), and Acid Alcohol Superfast Differentiation Solution (C0165M) were obtained from Beyotime (Shanghai, China). Stained sections were visualized using a light microscope, and representative images were acquired.

Enzyme-Linked Immunosorbent Assay

Hippocampal tissues were homogenized in ice-cold PBS (1:9, w/v) and centrifuged at 12000 × g for 10 minutes at 4°C. The supernatants were collected for quantification of Aβ40, Aβ42, TNF-α, IL-1β, and IL-6 using commercially available ELISA kits, according to the manufacturer's instructions. The ELISA kits for Aβ40 (JM-03151M1), Aβ42 (JM-11644M1), TNF-α (JM-02415M1), IL-1β (JM-02323M1), and IL-6 (JM-02446M1) were purchased from Jiangsu Jingmei Biotechnology Co., Ltd. (Jiangsu, China). Protein concentrations were determined using the BCA Protein Assay Kit (JM-9227A, Jiangsu Jingmei Biotechnology Co., Ltd.), and cytokine levels were normalized to the total protein concentration and expressed as pg/mg of protein.

Western Blot Analysis

Hippocampal tissue was lysed in RIPA buffer (1:9, w/v) and centrifuged at 12000 × g for 15 minutes at 4 °C. The supernatants were collected, and total protein concentrations were determined using the BCA assay. Equal amounts of protein were separated by SDS–PAGE and transferred to PVDF membranes. Membranes were blocked with 5% BSA in TBST for 2 h at room temperature and incubated overnight at 4 °C with the following primary antibodies: anti-C/EBPβ (1:1000, Affinity Biosciences, AF6202), anti-AEP (1:1000, Thermo Fisher Scientific, MA5-54040), anti-APP N-terminal (1:1000, Thermo Fisher Scientific, PA5-17829), anti-APP N585 (1:1000, Merck, ABN1642), anti-Tau5 (1:500, Thermo Fisher Scientific, AHB0042), anti-Tau N368 (1:1000, Merck, ABN1703), anti-phospho-Tau (Thr205) (1:1000, Cell Signaling Technology, 49561), anti-phospho-Tau (Ser396) (1:1000, Abcam, ab32057), and anti-β-actin (1:10000, Affinity Biosciences, T0022). After washing, membranes were incubated for 1 h at room temperature with HRP-conjugated secondary antibodies (1:10000, Affinity Biosciences, S0001). Signals were detected using an enhanced chemiluminescence (ECL) kit (PK10003, Wuhan Sanying) and visualized with a Tanon 5200 imaging system (Tanon, Shanghai).

Immunohistochemistry

Paraffin-embedded brain tissues were sectioned at 4–5 μm thickness. After dewaxing in xylene and rehydrating through a graded ethanol series, antigen retrieval was performed by microwaving the sections in citrate buffer (pH 6.0) for 5 minutes. Sections were cooled to room temperature and incubated with 3% hydrogen peroxide for 15 minutes to block endogenous peroxidase activity. Non-specific binding was blocked with 5% skimmed milk for 30 minutes at room temperature. Sections were then incubated overnight at 4°C with primary antibody against phosphorylated Tau (p-Tau, 1:250 dilution, Affinity Biosciences, AF3148). After washing with PBS, slides were incubated with HRP-conjugated secondary antibody (Goat Anti-Rabbit IgG (H+L) HRP, 1:200 dilution, Affinity Biosciences, S0001) at 37°C for 1 hour. Color development was carried out using DAB substrate (1:20 dilution, Super Plus™ Ultra-Sensitive IHC Detection Kit, Wuhan Elabscience, E-IR-R221), and the reaction was stopped with distilled water. Slides were counterstained with hematoxylin, dehydrated, cleared, and mounted with neutral balsam. Images were acquired using a light microscope.

Immunofluorescence Staining

Free-floating brain sections (25 μm) were washed in PBS and incubated with 0.3% hydrogen peroxide for 10 min to quench endogenous peroxidase activity. After three PBS washes, sections were blocked in 1% bovine serum albumin (BSA) with 0.3% Triton X-100 for 30 min at room temperature. Sections were then incubated overnight at 4 °C with the following primary antibodies diluted in blocking buffer: anti-Aβ (1:400, Proteintech, 25524-1-AP) and anti-C/EBPβ (1:200, Proteintech, 66649-1-Ig). After washing, sections were incubated for 1 h at room temperature in the dark with species-specific secondary antibodies. Nuclei were counterstained with DAPI (Beyotime, C1006). Sections were mounted and visualized using a fluorescence microscope. Fluorescence signal intensity was quantified using ImageJ software.

qPCR Analysis

Total RNA was isolated from hippocampal tissue using the TransZol Up RNA extraction kit (ET111-01-V2, TransGen Biotech, Beijing, China) following the manufacturer’s instructions. RNA concentration and purity were determined using a nucleic acid analyzer, with OD260/280 ratios between 1.8 and 2.0. Reverse transcription was carried out using the EasyScript One-Step gDNA Removal and cDNA Synthesis Kit (AE311-03, TransGen Biotech), under the following conditions: 42 °C for 15 min and 85 °C for 5 min. qPCR was performed in a 20 μL reaction volume using a standard three-step cycling protocol: initial denaturation at 95 °C for 3 min, followed by 40–45 cycles of 95 °C for 5 s, 55 °C for 10 s, and 72 °C for 15 s. Each reaction was run in triplicate. Relative gene expression levels were calculated using the 2^–ΔΔCT method, with GAPDH as the internal control. Primer sequences were designed based on published literature^8-10^ and synthesized by Servicebio (Wuhan, China); sequences are listed in Table 2.

**Table 2. Primers for Various Indicators**

| **Gene name** | **Forward** | **Reverse** |
| --- | --- | --- |
| C/EBPβ(*Cebpb*) | ACCGGGTTTCGGGACTTGA | GTTGCGTAGTCCCGTGTCCA |
| AEP(*Lgmn*) | TGGACGATCCCGAGGATGG | TTGACGCTGTACCAGTCACC |
| GAPDH | CCTCGTCCCGTAGACAAAATG | GTGGATGATCTGGTAGGCGT |

16S rRNA Gene Sequencing

Following the 16S rRNA gene sequencing methodology outlined by Ye et al.^11^, genomic DNA was extracted from fecal samples using the Omega Mag-Bind Soil DNA Kit according to the manufacturer’s protocol. DNA concentration was measured using a NanoDrop spectrophotometer and diluted to 1 ng/μL with sterile water. The V3-V4 hypervariable regions of bacterial 16S rRNA were amplified using primers Forward: CCTAYGGGRBGCASCAG and Reverse: GGACTACNNGGGTATCTAAT. After preparing the PCR reaction mixture, pre-denaturation was performed at 98°C for 5 minutes, followed by the amplification cycles: 98°C for 1 minute, then 98°C for 10 seconds, 50°C for 30 seconds, and 72°C for 30 seconds, repeated for 30 cycles, with a final extension at 72°C for 5 minutes. The PCR products were separated by 2% agarose gel electrophoresis, with the target bands excised and purified using the Axygen Gel Extraction Kit. The purified PCR products were then mixed in equal proportions and further cleaned using the Qiagen Gel Extraction Kit. Library construction was performed with the Illumina TruSeq Nano DNA LT Library Prep Kit. A 1 μL aliquot of the library sample was assessed for quality on an Agilent Bioanalyzer using the Agilent High Sensitivity DNA Kit. Sequencing was carried out on an Illumina NovaSeq (PE250 paired-end sequencing) instrument. Sequence analysis was conducted using QIIME2 2022.11 software for taxonomic classification and detailed data for α-diversity indices. Principal Coordinate Analysis (PCoA) plots were generated using R programming language.

Statistical Analysis

All statistical analyses were performed using GraphPad Prism 10. Data are presented as mean ± standard error of the mean (SEM). The normality of data distribution was assessed using the Shapiro–Wilk test. For normally distributed data, comparisons among multiple groups were made using one-way analysis of variance (ANOVA) followed by Dunnett’s post hoc test. For non-normally distributed data, non-parametric analyses (Kruskal–Wallis test) were applied. Morris water maze training data (escape latency across days) were analyzed using two-way ANOVA with Bonferroni’s post hoc test. A P-value of < 0.05 was considered statistically significant.

References

1. Reagan-Shaw S, Nihal M, Ahmad N. Dose translation from animal to human studies revisited. *Faseb j.* 2008;22(3):659-661.

2. Liu B. Regulation mechanism of Buyang Huanwu Decoction effects on APP/PS1 mice neuronal vascular unit RAGE/LRP1 receptor system, Ph.D. dissertation, Heilongjiang University of Chinese Medicine; 2017. Available at: https://kns.cnki.net/kcms2/article/abstract?v=m6wOUVKyYs6l89zg-DEIfiPOj9fRbz-xC6bDF-pN8ArzU5Mq9lRaQmO4J1hk6fCCVSqm_gRGE-ggSuRiMNhcMaNl5yHgl0xQpaRGzZRFRq0pTwqar0fd-72rwGvND0ZyRgOFLlqI84-UtIcJidN6vHJB4Y2okAH6onu8K6MNbmNqte4WOLhY_ofFHb4ujjiD&uniplatform=NZKPT&language=CHS

3. Xu Y, Chen B, Yi J, et al. Buyang Huanwu Decoction alleviates cerebral ischemic injury through modulating caveolin-1-mediated mitochondrial quality control. *Front Pharmacol.* 2023;14:1137609.

4. Tong W, Leng L, Wang Y, et al. Buyang huanwu decoction inhibits diabetes-accelerated atherosclerosis via reduction of AMPK-Drp1-mitochondrial fission axis. *J Ethnopharmacol.* 2023;312:116432.

5. Chen B, Xu Y, Tian F, et al. Buyang Huanwu decoction promotes angiogenesis after cerebral ischemia through modulating caveolin-1-mediated exosome MALAT1/YAP1/HIF-1α axis. *Phytomedicine.* 2024;129:155609.

6. Bai X, Wang B, Cui Y, et al. Hepcidin deficiency impairs hippocampal neurogenesis and mediates brain atrophy and memory decline in mice. *J Neuroinflammation.* 2024;21(1):15.

7. You L, Yu PP, Dong T, et al. Astrocyte-derived hepcidin controls iron traffic at the blood-brain-barrier via regulating ferroportin 1 of microvascular endothelial cells. *Cell Death Dis.* 2022;13(8):667.

8. Matsuda T, Kido Y, Asahara S, et al. Ablation of C/EBPbeta alleviates ER stress and pancreatic beta cell failure through the GRP78 chaperone in mice. *J Clin Invest.* 2010;120(1):115-126.

9. Solberg R, Lunde NN, Forbord KM, Okla M, Kassem M, Jafari A. The Mammalian Cysteine Protease Legumain in Health and Disease. *Int J Mol Sci.* 2022;23(24).

10. Zhao P, He XB, Chen XY, et al. Celastrol inhibits mouse B16-F10 melanoma cell survival by regulating the PI3K/AKT/mTOR signaling pathway and repressing HIF-1α expression. *Discov Oncol.* 2024;15(1):178.

11. Ye Q, Sun S, Deng J, et al. Using 16S rDNA and metagenomic sequencing technology to analyze the fecal microbiome of children with avoidant/restrictive food intake disorder. *Scientific Reports.* 2023;13(1):20253.
